# Fetuin-A and risk of diabetes-related vascular complications: a prospective study

**DOI:** 10.1186/s12933-021-01439-8

**Published:** 2022-01-08

**Authors:** Anna Birukov, Elli Polemiti, Susanne Jäger, Norbert Stefan, Matthias B. Schulze

**Affiliations:** 1 Department of Molecular Epidemiology, German Institute of Human Nutrition Potsdam-Rehbruecke, Arthur-Scheunert-Allee 114-116, 14558 Nuthetal, Germany; 2 German Center for Diabetes Research (DZD), Munich-Neuherberg, Germany; 3 Department of Internal Medicine IV, University Hospital of Tübingen, Tübingen, Germany; 4 Institute of Diabetes Research and Metabolic Diseases of the Helmholtz Center Munich, Tübingen, Germany; 5 Institute of Nutritional Science, University of Potsdam, Nuthetal, Germany

**Keywords:** Fetuin-A, Biomarkers, Epidemiology, Type 2 diabetes, Vascular disease, Vascular calcification, Microvascular complications

## Abstract

**Background:**

Fetuin-A is a hepatokine which has the capacity to prevent vascular calcification. Moreover, it is linked to the induction of metabolic dysfunction, insulin resistance and associated with increased risk of diabetes. It has not been clarified whether fetuin-A associates with risk of vascular, specifically microvascular, complications in patients with diabetes. We aimed to investigate whether pre-diagnostic plasma fetuin-A is associated with risk of complications once diabetes develops.

**Methods:**

Participants with incident type 2 diabetes and free of micro- and macrovascular disease from the European Prospective Investigation into Cancer and Nutrition (EPIC)-Potsdam cohort (n = 587) were followed for microvascular and macrovascular complications (n = 203 and n = 60, respectively, median follow-up: 13 years). Plasma fetuin-A was measured approximately 4 years prior to diabetes diagnosis. Prospective associations between baseline fetuin-A and risk of complications were assessed with Cox regression.

**Results:**

In multivariable models, fetuin-A was linearly inversely associated with incident total and microvascular complications, hazard ratio (HR, 95% CI) per standard deviation (SD) increase: 0.86 (0.74; 0.99) for total, 0.84 (0.71; 0.98) for microvascular and 0.92 (0.68; 1.24) for macrovascular complications. After additional adjustment for cardiometabolic plasma biomarkers, including triglycerides and high-density lipoprotein, the associations were slightly attenuated: 0.88 (0.75; 1.02) for total, 0.85 (0.72; 1.01) for microvascular and 0.95 (0.67; 1.34) for macrovascular complications. No interaction by sex could be observed (p > 0.10 for all endpoints).

**Conclusions:**

Our data show that lower plasma fetuin-A levels measured prior to the diagnosis of diabetes may be etiologically implicated in the development of diabetes-associated microvascular disease.

**Supplementary Information:**

The online version contains supplementary material available at 10.1186/s12933-021-01439-8.

## Background

Fetuin-A is a hepatic secretory glycoprotein that on the one hand promotes insulin resistance by inhibiting the insulin receptor tyrosine kinase in skeletal muscle and hepatocytes, serving as an adaptor protein for saturated fatty acids and allowing them to activate Toll-like receptor 4 (TLR4), thereby inducing inflammatory signaling and insulin resistance [1, 2]. Another possible link between fetuin-A and insulin resistance is fetuin-A-stimulated inflammation in pancreatic adipocytes and islets, and fetuin-A-mediated c-Jun N-terminal kinase- and Ca-dependent impairment of insulin secretion [3, 4]. On the other hand, fetuin-A inhibits soft tissue calcification [5–7] through binding of small clusters of calcium and phosphate, preventing their growth, aggregation and mineral precipitation, stabilizing these ions and preventing their uptake by cells [4, 7, 8]. These soluble protein-mineral colloids known as calciprotein particles are subsequently cleared by the reticuloendothelial system. In addition, fetuin-A enhances phagocytosis of extracellular vesicles and apoptotic cells by vascular smooth muscle cells and macrophages, reducing both apoptosis and calcification in cells subjected to elevated extracellular concentrations of mineral ions [7]. In advanced chronic kidney disease (CKD) and cardiovascular disease (CVD), fetuin-A inhibits ectopic and dystrophic calcium deposition [5, 9–13], and in mice, fetuin-A shows cardio- and vasculoprotective properties through reduced calcification [5–7].

Human prospective studies on fetuin-A and incidence of diabetes or CVD have so far yielded contradicting results [14–23]. Although several systematic reviews reported an increased risk of type 2 diabetes (T2D) with higher fetuin-A concentrations [14, 24, 25], two recent Mendelian Randomization (MR) studies did not support the causality of these associations [16, 17]. Fetuin-A was positively associated with CVD in healthy community-dwelling adults in EPIC-Potsdam [19], a finding which was subsequently corroborated by a genetic analysis [18]. However, a recent meta-analysis of 7 prospective studies (exclusive EPIC-Potsdam) could not confirm a higher CVD risk for genetic variants associated with higher fetuin-A levels [20]. As to diabetes complications, the previous studies were primarily of cross-sectional nature and reported inverse associations of fetuin-A with macrovascular events, such as peripheral arterial disease [26, 27] and the presence of atherosclerotic plaques [28] in individuals with T2D. The only two prospective studies on fetuin-A and risk of macrovascular diabetes-associated disease are the Rancho Bernardo Study (RBS) and Cardiovascular Health Study (CHS) studies, in which higher baseline fetuin-A levels among individuals with diabetes were related to higher rates of cardiovascular morbidity and mortality [21, 22]. The conflicting data on fetuin-A regulation in cardiometabolic disease onset clearly need to be reconciled in terms of timing of events and causality. Yet prospective epidemiologic studies to evaluate associations with microvascular outcomes in diabetes are still missing. Moreover, no study so far has examined the relationships of vascular diabetes-related complications with plasma fetuin-A measured prior to diabetes diagnosis and unaffected by the presence of disease or treatment, which is key for etiological research. In this prospective study we investigated the relationships between circulating pre-diagnosis fetuin-A concentrations and risk of diabetes-related microvascular and macrovascular disease, controlling for a wide range of potential confounders and across both sexes and subgroups with different liver and kidney functions, BMI, fasting state, and glucose metabolism.

## Methods

### Study design and population

**Fig. 1:**
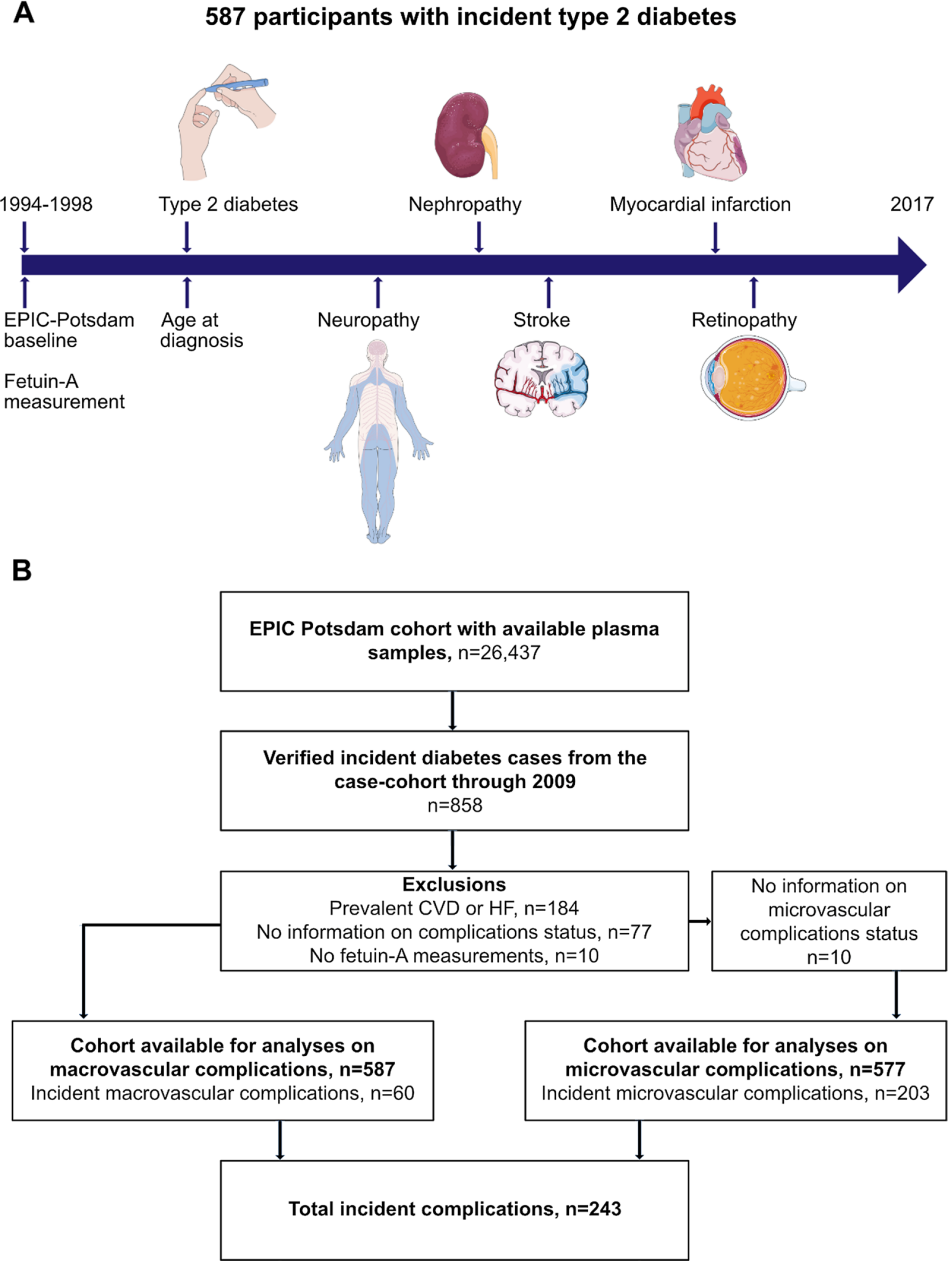
Study design and flow chart of inclusion. **A** For the present study, the follow-up time was defined as the time between type 2 diabetes diagnosis and diagnosis of the corresponding vascular disease or date of the last examination by the physicians (up until August 2017). **B** Microvascular complications were defined as new-onset retinopathy, nephropathy, neuropathy or kidney replacement therapy following diabetes diagnosis. Macrovascular complications were defined as newly diagnosed myocardial infarction or stroke following diabetes diagnosis. EPIC, European Prospective Investigation into Cancer and Nutrition; CVD, cardiovascular disease; HF, heart failure

The study is embedded in the European Prospective Investigation Into Cancer and Nutrition (EPIC)-Potsdam cohort, initially comprising 27,548 individuals (16,644 women aged 35–65 years and 10,904 men aged 40–65 years) with available blood sample measurements [29]. Participants were recruited between 1994 and 1998 from the general population of Potsdam area, Germany. At the time of recruitment (hereafter called “baseline”), anthropometric and blood pressure measurements were taken, blood samples were drawn, followed by an interview and a questionnaire on prevalent medical conditions, sociodemographic and lifestyle characteristics. Follow-up on incident T2D and CVD, diet and other lifestyle factors is conducted every 2–3 years. The analytical sample consisted of all incident T2D cases identified by December 2009 with available fetuin-A measurements at baseline and follow-up information on complications status from the time of diabetes diagnosis through August 2017 (n = 587), Fig. 1A, B.

### Case ascertainment

Standardized forms were sent to the treating physicians of all incident diabetes cases identified in EPIC-Potsdam, retrospectively assessing the diagnosis of micro- and macrovascular complications from the time of diabetes diagnosis until 2017, Fig. 1A. Complications were considered incident if diagnosed after diabetes onset. Microvascular complications comprised diabetic kidney disease (ICD-10 E11.2; including unspecified diabetes-related nephropathy and renal replacement therapy or albuminuria), retinopathy (ICD-10 E11.3, including proliferative or non-proliferative retinopathy, or blindness) and neuropathy (ICD-10 E11.4, including unspecified diabetes-related peripheral neuropathy, loss of sensation or diabetic foot syndrome). Macrovascular complications encompassed myocardial infarction (ICD-10 I21), stroke (ischemic ICD-10 I63.0–I63.9, hemorrhagic ICD-10 I60.0–I61.9 and undetermined stroke ICD-10 I64.0–I64.9) after diabetes diagnosis and amputation due to diabetic foot ulcers. Systematic information sources for incident diabetes were self-report of a respective diagnosis, pharmacological treatment or dietary change due to disease, collected through regular follow-up questionnaires. Further sources of information included death certificates, tumor centers, clinical records linkage. Verification of potential diabetes cases was carried out through standardized forms filled by the participants’ treating physicians containing the date and type of diagnosis, the diagnostic tests used (several abnormal fasting blood glucose readings, several abnormal postprandial blood glucose readings, abnormal HbA1c, abnormal urine glucose) and the methods of treatment. The majority of diagnoses were based on a combination of above-mentioned diagnostic tests, only 4% (22/587) were diagnosed based on abnormal HbA1c or urine glucose alone, and 12 participants were lacking information on the applied diagnostic test. Only physician-verified cases with a diagnosis date after the baseline examination were considered confirmed incident cases of T2D. Incident macrovascular events were also ascertained from the regular follow-up of participants, following the same procedure as diabetes ascertainment.

### Laboratory analyses

A sample of 30 ml blood was drawn from mainly non-fasting participants at baseline and collected in monovettes containing 10% citrate. Samples were aliquoted and stored in tanks of liquid nitrogen (− 196 °C) or deep freezers (− 80 °C). Analyses of all biomarkers were performed at the Department of Internal Medicine, University of Tübingen in 2007 and 2008. Plasma levels of high-density lipoprotein (HDL) and total cholesterol, hemoglobin A_1c_ (HbA_1c_), triglycerides, high-sensitivity CRP (CRP), creatinine and fetuin-A were measured with the automatic ADVIA 1650 analyzer (Siemens Medical Solutions, Erlangen, Germany). Adiponectin was measured with an enzyme-linked immunosorbent assay from Linco Research, St Charles, MO, USA. Fetuin-A was determined with an immunoturbidimetric method with specific polyclonal goat anti-human fetuin-A antibodies to human fetuin-A (BioVendor Laboratory Medicine, Modreci, Czech Republic). This method was evaluated in a side-by-side comparison with an ELISA (intra-assay coefficient of variation, 3.5%; inter-assay coefficient of variation, 5.4%; BioVendor) showing a correlation of r = 0.93 [19]. Since in EPIC-Potsdam all blood biomarkers were measured in citrate plasma, they were corrected for the dilution introduced by citrate volume (multiplied by 1.16 for women and 1.17 for men) to obtain concentrations comparable to concentrations measured in EDTA plasma. These factors were based on the hypothesis that, on average, the hematocrit was 0.44 in men and 0.40 in women—similar to the mean hematocrit observed in the German National Health Interview and Examination Survey in 1998 (Robert Koch Institute, unpublished data)—and that, after centrifugation of the blood with anticoagulant, all citrate was in the plasma [30].

### Statistical analyses

In the participants who subsequently developed diabetes, characteristics were evaluated according to the quintiles of baseline fetuin-A concentrations. Missing data were not imputed, participants with missing data were excluded from the respective analyses. Concentrations of triglycerides, CRP, HbA_1c_ and adiponectin were non-normally distributed, values of these biomarkers were normalized by natural log-transformation prior to any analyses. Correlation between fetuin-A and lifestyle and cardiometabolic risk factors (BMI, blood pressure, blood lipids, glycemia, markers of inflammation, kidney function etc.) was assessed with age- and sex-adjusted Spearman correlation coefficients.

For associations between baseline fetuin-A concentrations and micro- and macrovascular complications, Cox regression models stratified by age at diabetes diagnosis were applied. To account for the duration of diabetes, participant’s age was used as underlying timescale, with entry time as age at diabetes diagnosis and exit time as age at event or censoring (date of last examination by the treating physician). We additionally adjusted for the duration between baseline and diabetes diagnosis in all statistical models. The associations were assessed on a continuous scale, by one standard deviation (SD) increase in baseline fetuin-A concentration. Basic multivariable model (model 1) was adjusted for the duration between recruitment and diabetes diagnosis, sex, education (three categories: no or in vocational training, vocational training/technical school, technical college or university), alcohol intake (six categories: < 6.1 g/day, 6.1–12.0 g/day, 12.1–24.0 g/day, 24.1–60.0 g/day, 60.1–96.0 g/day, > 96.0 g/day), smoking (four categories: never smoker, former smoker, current smoker < 20 cigarettes/day, current heavy smoker ≥ 20 cigarettes/day), physical activity (sports ≤ 4 h/week, sports > 4 h/week, biking < 2.5 h/week, biking 2.5–4.9 h/week, biking ≥ 5 h/week), BMI, waist circumference, history of hypertension, hyperlipidemia, antihypertensive and lipid lowering medications at the time of recruitment. Model 2 was additionally adjusted for baseline estimated glomerular filtration rate (eGFR), CRP, non-HDL cholesterol, HbA_1c_ and adiponectin. eGFR was estimated with CKD-EPI formula. The final model 3 was further adjusted for baseline triglycerides and HDL-cholesterol concentrations. The shape of association with all outcomes was evaluated with restricted cubic splines with 3 knots at 5th, 50th and 95th percentiles, applied on the basic model. Median fetuin-A concentration (0.28 g/L) was used as the reference point. Non-linear trend was assessed with the Wald test. We performed several sensitivity analyses: excluding individuals with baseline HbA_1c_ ≥ 6.5%, including individuals with incident vascular complications prior to diabetes diagnosis, stratifying by sex, baseline eGFR, fasting status, fatty liver status, elevated glucose (i.e., ≥ 100 mg/dL) at baseline and complication burden during follow-up. Fatty liver index (FLI) was calculated applying the formula by Bedogni et al. [31], which can be found in the Additional file 1: Expanded Materials and Methods.

We further assessed whether the associations differed by type of microvascular event (nephropathy, neuropathy, retinopathy), censoring at first respective event. Interaction between fetuin-A and sex and all other above-listed covariates was tested by creating cross product terms and evaluating the significance level. Because there is evidence that several dietary factors including coffee, dairy intake and omega-3 fatty acids are associated with circulating fetuin-A levels (for review see Icer and Yıldıran [32]), we also adjusted our analyses for these factors in sensitivity analyses.

Finally, we examined the associations between the single nucleotide polymorphism (SNP) rs4917 of the fetuin-A encoding gene *AHSG,* which has the strongest association with circulating fetuin-A concentrations [18], and risk of diabetes-associated vascular complications. These associations were assessed by Cox proportional hazard model and adjusted for sex and age (underlying time scale). Details about genotyping methods can be found in the Additional file 1: Expanded Materials and Methods.

A two-sided p < 0.05 denoted statistical significance in all analyses. All statistical analyses were performed using SAS (Version 9.4, Enterprise Guide 7.1, SAS Institute Inc., Cary, NC, USA).

## Results

### Participants’ characteristics

Clinical and demographic characteristics of the participants with incident diabetes are presented in Table 1 according to the quintiles of baseline plasma fetuin-A concentrations. Percentage of women and obesity as well as HDL-cholesterol concentrations increased, while physical activity level decreased across fetuin-A quintiles. There was a tendency to lower circulating creatinine and higher CRP with increasing fetuin-A. Fetuin-A correlated weakly directly with HDL-cholesterol (r = 0.14), non-HDL cholesterol (r = 0.08) and HbA_1c_ (r = 0.10), and BMI/waist circumference (r = 0.08), and inversely with physical activity (r = -0.12), after adjustment for age and sex, Additional file 1: Table S1. Participants who developed vascular complications of diabetes were on average older, more likely to be men and had a more unfavorable cardiometabolic profile characterized by higher triglycerides, lower HDL-cholesterol and lower adiponectin [33]. The median follow-up time (IQR) from diabetes diagnosis on was 12.8 (10.5–15.1) years for microvascular complications and 13.4 (10.9–15.6) years for macrovascular complications.

**Table 1:** Participants’ characteristics according to quintiles of fetuin-A, n = 587

Fetuin-A [g/L]	1st quintile	2nd quintile	3rd quintile	4th quintile	5th quintile	Total
	0.20 (0.04)	0.24 (0.02)	0.28 (0.01)	0.31 (0.02)	0.35 (0.04)	0.28 (0.08)
Sociodemographics						
Age at recruitment, y	55.0 (12.0)	55.5 (10.0)	56.0 (11.0)	55.0 (11.0)	54.0 (14.0)	55.0 (12.0)
Age at T2D diagnosis, y	59.2 (11.8)	60.4 (10.2)	59.2 (10.6)	57.8 (12.5)	58.3 (13.2)	59.0 (11.8)
Duration of T2D, y	11.8 (5.35)	12.2 (4.61)	12.3 (4.78)	12.5 (5.43)	13.5 (4.70)	12.4 (5.17)
Women, n (%)	40 (34.2)	48 (40.7)	53 (45.3)	49 (41.9)	69 (58.5)	259 (44.1)
BMI, kg/m^2^	29.1 (4.92)	29.8 (5.82)	30.4 (4.90)	29.8 (6.06)	30.5 (6.94)	29.9 (5.76)
Obesity, n (%)	44 (37.6)	56 (47.5)	63 (53.9)	57 (48.7)	67 (56.8)	287 (48.9)
Higher education (university or technical college), n (%)	38 (32.5)	39 (33.1)	41 (35.0)	35 (29.9)	30 (25.4)	183 (31.2)
Physical activity, h/week	1.52 (0.32)	1.47 (0.32)	1.47 (0.30)	1.46 (0.28)	1.42 (0.24)	1.47 (0.29)
Alcohol consumption, g/day	11.1 (23.1)	8.18 (17.4)	9.67 (20.5)	9.25 (25.0)	6.32 (12.0)	8.36 (18.5)
Smoking						
Former smoker, n (%)	40 (34.2)	56 (47.5)	47 (40.2)	60 (51.3)	51 (43.2)	254 (43.3)
Current smoker < 20 cigarettes/day	19 (16.2)	12 (10.2)	15 (12.8)	8 (6.8)	13 (11.0)	67 (11.4)
Current smoker ≥ 20 cigarettes/day	16 (13.7)	11 (9.3)	12 (10.3)	11 (9.4)	7 (5.9)	57 (9.7)
Prevalent hypertension, n (%)	86 (73.5)	85 (72.0)	85 (72.7)	88 (75.2)	86 (72.9)	430 (73.3)
Prevalent hyperlipidemia, n (%)	48 (41.0)	40 (33.9)	49 (41.9)	48 (41.0)	52 (44.1)	237 (40.4)
Fatty liver index	75.5 (47.6)	78.4 (40.0)	76.5 (34.8)	81.7 (34.1)	79.1 (34.9)	78.2 (38.1)
eGFR, mL/min/1.73m^2^	92.8 (18.4)	88.8 (20.7)	89.3 (21.0)	90.6 (20.5)	92.3 (21.5)	90.9 (20.4)
Biomarkers						
HbA_1c_, %	5.97 (0.83)	6.17 (1.06)	6.15 (1.01)	6.18 (1.01)	6.16 (1.14)	6.12 (1.03)
HbA_1c_, mmol/mol	41.7 (2.91)	43.9 (3.12)	43.7 (3.07)	44.0 (3.07)	43.8 (3.19)	43.4 (11.3)
Total cholesterol, mg/dL	205 (68.1)	209 (56.9)	220 (46.9)	216 (47.0)	212 (47.0)	213 (52.3)
Non-HDL-cholesterol, mg/dL	158 (65.7)	161 (53.7)	168 (36.6)	168 (44.9)	167 (42.8)	166 (46.3)
Triglycerides, mg/dL	180 (215)	165 (92.8)	168 (116)	180 (106)	167 (103)	170 (123.2)
HDL-cholesterol, mg/dL	42.2 (15.3)	45.6 (13.6)	46.2 (12.3)	47.2 (12.2)	47.7 (12.3)	46.1 (13.7)
CRP, mg/dL	0.15 (0.32)	0.19 (0.28)	0.21 (0.43)	0.20 (0.31)	0.23 (0.43)	0.19 (0.36)
Creatinine, mg/dL	0.87 (0.22)	0.87 (0.26)	0.86 (0.23)	0.84 (0.27)	0.81 (0.20)	0.85 (0.25)
Adiponectin, µg/mL	5.07 (3.52)	5.25 (2.85)	5.54 (3.34)	5.35 (3.29)	5.70 (3.82)	5.37 (3.35)

Data are presented as median values (interquartile range) at recruitment, if not otherwise stated. BMI = body mass index, HbA_1c_ = hemoglobin A_1c_, HDL = high density lipoprotein, CRP = high sensitivity reactive protein C, eGFR = estimated glomerular filtration rate estimated with Chronic Kidney Disease Epidemiology Collaboration group (CKD-EPI) formula. Obesity was defined as BMI ≥ 30 kg/m^2^

### Associations between fetuin-A and vascular complications

**Fig. 2:**
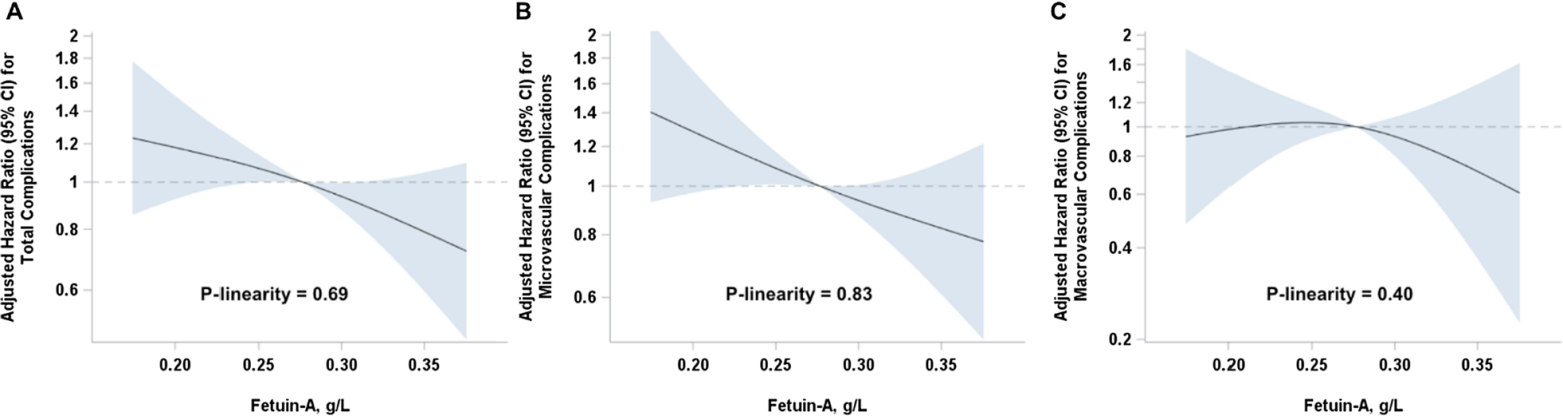
Associations of pre-diagnosis fetuin-A concentrations with risks of diabetes-related vascular complications. Restricted cubic splines showing the shape of dose–response curve according to baseline fetuin-A concentration: hazard ratios (solid lines) with corresponding 95% CI (shaded area) for total vascular (**A**), microvascular (**B**), and macrovascular complications (**C**). The reference point is median fetuin-A concentration with knots placed at the 5th, 50th, and 95th percentiles. All models were adjusted for age at diabetes diagnosis (underlying time scale), duration between recruitment and diabetes diagnosis, sex, education, alcohol intake, physical activity, BMI, waist circumference, history of hypertension, hyperlipidemia, antihypertensive and lipid lowering medications at the time of recruitment

**Fig. 3:**
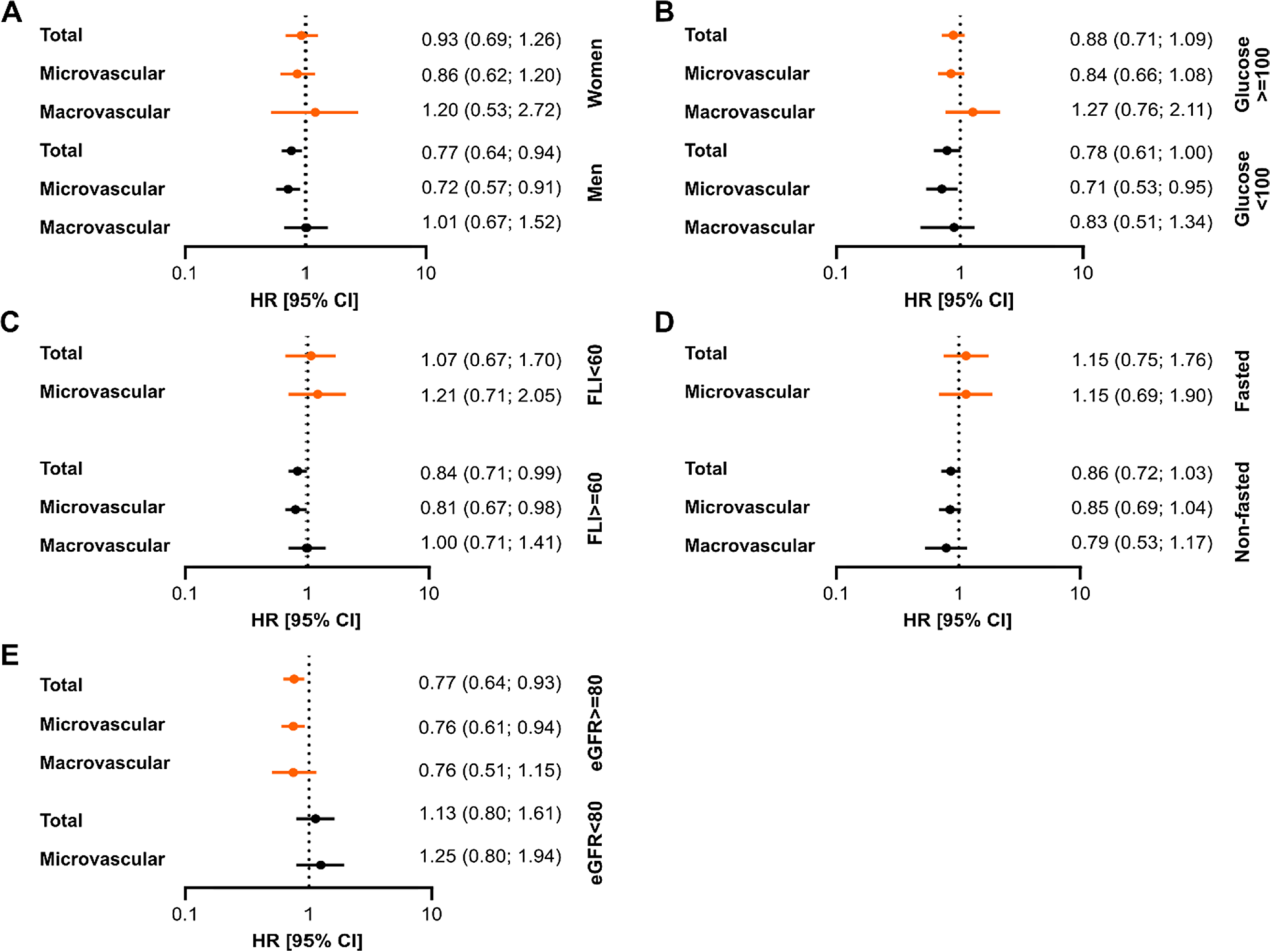
Stratified associations between pre-diagnostic fetuin-A and incident diabetes-related complications. **A** P for interaction with sex p = 0.18 for complications overall, p = 0.18 for microvascular and p = 0.56 for macrovascular complications. **B** P for interaction with baseline glucose (glucose ≥ 100 mg/dL vs glucose < 100 mg/dL) p = 0.85 for complications overall, p = 0.67 with microvascular and p = 0.31 for macrovascular complications. **C** P for interaction with FLI (FLI < 60 vs FLI ≥ 60) p = 0.94 for complications overall, p = 0.65 for microvascular complications. **D** P for interaction with baseline fasting status (fasted vs non-fasted) p = 0.36 for complications overall and p = 0.57 with microvascular complications. **E** P for interaction with baseline eGFR (eGFR ≥ 80 mL/min/1.73 m^2^ vs < 80 mL/min/1.73 m^2^) p = 0.10 for complications overall, p = 0.15 for microvascular complications. There were not enough macrovascular events in the subgroups with baseline eGFR < 80 mL/min/1.73 m^2^, FLI < 60 and fasted subgroup to perform the analysis. Associations were assessed by Cox proportional hazards models and are shown per one unit SD increase (0.06 g/L) in pre-diagnostic fetuin-A concentrations. Models are adjusted for age at diabetes diagnosis (underlying time scale), duration between recruitment and diabetes diagnosis, sex, education (three categories: no or in vocational training, vocational training/technical school, technical college or university), alcohol intake (six categories: < 6.1 g/day, 6.1–12.0 g/day, 12.1–24.0 g/day, 24.1–60.0 g/day, 60.1–96.0 g/day, > 96.0 g/day), smoking (four categories: never smoker, former smoker, current smoker < 20 cigarettes/day, current heavy smoker ≥ 20 cigarettes/day), physical activity (sports ≤ 4 h/week, sports > 4 h/week, biking < 2.5 h/week, biking 2.5–4.9 h/week, biking ≥ 5 h/week), body mass index, waist circumference, history of hypertension, hyperlipidemia, antihypertensive and lipid lowering medications at the time of recruitment. HR, hazard ratio; CI, confidence interval; SD, standard deviation; eGFR, estimated glomerular filtration rate; FLI, fatty liver index

In multivariable Cox regression analyses, lower baseline fetuin-A concentrations were linearly associated with higher risk of total and microvascular complications, Fig. 2 and Table 2. Each 0.06 g/L (one SD) increase in baseline fetuin-A concentration was associated with a 14% lower risk of any diabetes complications (HR 0.86, 95% CI 0.74; 0.99) and 16% lower risk of microvascular complications (HR 0.84, 95% CI 0.71; 0.98) in the basic model, Table 2. There was no significant association with macrovascular endpoints (HR 0.92, 95% CI 0.68; 1.24). Further adjustment for CRP, non-HDL cholesterol, HbA_1c_, adiponectin and eGFR did not substantially alter the estimates (Table 2, Model 2), however, the adjustment for triglycerides and HDL-cholesterol slightly attenuated the results to 0.88 (0.75; 1.02) for any diabetes complications, 0.85 (0.72; 1.01) for microvascular and 0.95 (0.67; 1.34) for macrovascular complications. The relationships did not differ by sex, glucose, FLI categories or fasting status, Fig. 3A–D. Even though the inverse associations with fetuin-A seemed to be pronounced only in individuals with eGFR ≥ 80 mL/min/1.73 m^2^, the interaction analysis did not support a significant effect modification by baseline kidney function (p = 0.10 for complications overall, p = 0.15 for microvascular complications, the analysis for macrovascular complications was not possible due to low numbers, Fig. 3E). No interaction with other covariates could be detected for any of the vascular endpoints either, all p > 0.05 (data not shown).

**Table 2:** Adjusted hazard ratios for diabetes-related vascular complications per SD increase in baseline fetuin-A concentrations

	Total vascular complications		Microvascular complications		Macrovascular complications	
	N cases/total N	HR (95% CI) per 1 SD (0.06 g/L) difference in fetuin-A	N cases/total N	HR (95% CI) per 1 SD (0.06 g/L) difference in fetuin-A	N cases/total N	HR (95% CI) per 1 SD (0.06 g/L) difference in fetuin-A
Model 1	243/587	0.86 (0.74; 0.99)	203/577	0.84 (0.71; 0.98)	60/587	0.92 (0.68; 1.24)
Model 2	232/556	0.85 (0.73; 0.99)	195/546	0.83 (0.70; 0.98)	56/556	0.89 (0.64; 1.24)
Model 3	232/556	0.88 (0.75; 1.02)	195/546	0.85 (0.72; 1.01)	56/556	0.95 (0.68; 1.34)

HR, hazard ratio; CI, confidence interval

Even lower risk per SD increase in circulating fetuin-A levels was observed after exclusion of participants with baseline HbA_1c_ ≥ 6.5%, HR 0.80 (95% CI 0.67; 0.97) for total and 0.76 (0.61; 0.95) for microvascular complications, Additional file 1: Table S2. The associations did not differ by the type of microvascular complication, HR 0.90 (95% CI 0.70; 1.17) for nephropathy, 0.84 (0.65; 1.08) for neuropathy and 0.91 (0.38; 2.18) for retinopathy occurring as first event, Additional file 1: Table S3. We also examined whether the associations between baseline fetuin-A and incidence of vascular complications differed by complication load. The relationships seemed to be stronger in individuals with less severe complication burden: HR per SD 0.82 (95% CI 0.69; 0.98) among participants with one complication as opposed to 0.94 (0.73; 1.23) among those with more than one complication during the follow-up, Additional file 1: Table S4. Inclusion of participants with incident vascular comorbidities prior to the diagnosis of diabetes (n = 25) slightly attenuated the estimates: 0.91 (0.80; 1.04) for total vascular complications, 0.89 (0.76; 1.03) for microvascular and 0.94 (0.75; 1.18) for macrovascular complications, Additional file 1: Table S5. Adjustment for daily coffee, dairy intake or circulating omega-3 fatty acids and palmitic acid did not appreciably alter the associations, Additional file 1: Table S6.

### Genetic associations

The *AHSG* SNP rs4917 explained 17% of the phenotypic variation in fetuin-A levels in this population. No significant association between rs4917 and risk of complications was observed, HR (95% CI) per increase in C-allele: 1.08 (0.88; 1.33) for total, 1.06 (0.84; 1.33) for microvascular and 1.13 (0.74; 1.71) for macrovascular complications (data not tabled).

## Discussion

### Main findings

In the present study, pre-diagnosis plasma fetuin-A was inversely associated with the risk of incident total vascular, notably microvascular, complications among individuals with T2D, independent of major cardiometabolic risk factors. The relationships were slightly attenuated after correcting for baseline triglycerides and HDL-cholesterol concentrations. We observed a similar magnitude of the associations with individual microvascular endpoints nephropathy, neuropathy, and retinopathy, though these relationships failed to reach statistical significance.

### Possible biological mechanisms

One possible mechanism for the protective properties of fetuin-A against the development of diabetes-related vascular complications may be its potent inhibitory role in pathological vascular mineralization process [5–8]. In patients with coronary heart and aortic valve disease, fetuin-A inversely associates with aortic and mitral annular calcification and aortic stenosis [9, 11]. In patients with CKD, higher serum fetuin-A levels are associated with lower risk of all-cause and cardiovascular mortality [10, 34], and with decreased mineral crystal maturation, osteochondrogenic differentiation and inflammatory processes in the extracellular vesicles, thereby suggesting an increased calcification inhibitory capacity with higher fetuin-A levels in CKD [12]. In the EPIC-Potsdam cohort, participants were disease-free at baseline with a latency period of approximately 15 years between blood draw and the development of complications, and the protective effects of fetuin-A were more prominently seen in individuals with a “healthier” phenotype (baseline eGFR ≥ 80 mL/min/1.73 m^2^, lower baseline HbA_1c_ levels, less severe complication burden during the follow-up)suggesting that fetuin-A might contribute to blood and tissue mineral homeostasis also in physiological conditions.

### Findings in light of other evidence

Our data are congruent with three cross-sectional studies in patients with diabetes showing inverse associations between fetuin-A and peripheral arterial disease [26, 27], and the prevalence of atherosclerotic calcified plaques [28]. In a prospective study assessing factors associated with lower limb calcification progression in T2D, baseline fetuin-A levels were inversely correlated to this progression only in univariate but not in multivariable analyses, potentially because the contribution of fetuin-A was fully captured through the mediator pathway—baseline calcification score—which the authors corrected for in fully adjusted models [35]. In contrast to our observation, positive associations of fetuin-A with CVD risk in individuals with prevalent diabetes were reported from the RBS and CHS studies [21, 22]. The EPIC-Potsdam participants differed in two aspects from RBS and CHS. First, they were substantially younger at blood draw (median age 55 years) and at the diabetes diagnosis (median age 59 years) than the participants in RBS or CHS (mean age 73 and 75 years, respectively). Second, fetuin-A was measured before diabetes onset in EPIC-Potsdam, but in overt diabetes in RBS and CHS. The manifestation of metabolic abnormalities in combination with advanced age could confound the relationships between circulating fetuin-A and vascular disease in RBS and CHS. E.g. in the presence of progressed metabolic perturbations and chronic low-grade inflammation, fetuin-A could exacerbate insulin resistance and worsen the proatherogenic milieu. The low number of macrovascular diabetes-related complications in the EPIC-Potsdam study precludes any definitive conclusions regarding the biological involvement of fetuin-A in CVD secondary to T2D.

### Subgroup and genetic analyses

Fetuin-A has been reported to exert both pro- and anti-inflammatory responses. On the one hand, fetuin-A acts as an endogenous ligand for the TLR4, which enables free fatty acids to activate TLR4-signaling to induce insulin resistance, and stimulates production of pro-inflammatory cytokines from adipocytes and macrophages [1]. The positive association between fetuin-A and insulin resistance is supported by epidemiologic studies [2, 36] and in mice, injection of recombinant fetuin-A decreases insulin sensitivity, *Ahsg*^*−/−*^ mice are insulin-sensitive and resistant to weight gain when fed a high-fat diet [37]. On the other hand, fetuin-A is essential for the inhibition of the pro-inflammatory cytokine tumor necrosis factor and the NLRP3 inflammasome in macrophages, TGF-β1 antagonization and regulation of macrophage polarization [7, 38]. We did not observe any statistically significant relationship between the inflammatory marker CRP or adiposity measures and fetuin-A, which is in line with previously published data from EPIC-Potsdam [15]. Fetuin-A has also been found to accelerate incorporation of exogenous fatty acids into cellular triglycerides and cholesterol efflux from cells [7], yet no substantial correlation between fetuin-A and cholesterol or triglycerides could be observed in our study, and the adjustment for these confounders did not essentially alter the estimates. Some studies suggested increased levels of fetuin-A in NAFLD [36], inverse [39, 40] or positive associations [41, 42] between NAFLD and vascular complications in diabetes patients. We did not detect any effect modification by FLI, and fetuin-A only weakly correlated with FLI. In the previous study from EPIC-Potsdam, fetuin-A was associated with increased diabetes risk particularly in individuals with elevated plasma glucose [15], which was in agreement with a negative relationship of plasma fetuin-A levels with insulin secretion in participants with impaired glucose tolerance but not in participants with normal glucose tolerance [43]. Yet no modification by plasma glucose levels could be observed for the risk of diabetes-related complications in the present study, as in the previous study by Weikert et al. for the risk of CVD [19].

Among individuals with diabetes in EPIC-Potsdam, there was no association between *AHSG* SNP rs4917 and risk of microvascular complications, though we might have been underpowered to detect such relationship. There was a tendency towards lower risk of diabetic retinopathy per minor allele (T) increase in rs4917 in African Americans in a recent GWAS by Pollack et al. [44], which became significant after pooling with genotyping data from Europeans [45]. However, in FinnGen Study, the SNP rs4917 was not significantly associated with diabetic retinopathy [46].

### Strengths and limitations

The strengths of this study are long follow-up period, relatively large sample size, use of physician-validated diagnoses, high response rate of questionnaires from physicians and a use of both micro- and macrovascular diabetes endpoints in the same study. A particular advantage of this study are fetuin-A measurements at recruitment, relatively long before diabetes diagnosis, which is key for the detection of potential causality in the associations between fetuin-A levels and the development of diabetes complications, since the concentrations are not influenced by pathophysiological processes of overt disease and treatment in the present study. Several limitations also need to be mentioned. First, no direct measures of vascular calcification were available in our study, thus we can only speculate about potential mechanisms involving inhibition of vascular calcification that link higher fetuin-A to lower risk of microvascular disease in diabetes. The number of macrovascular complications was relatively low in our study; thus, we did not have sufficient statistical power to examine the associations with CVD secondary to diabetes. Fetuin-A was measured approximately 15 years prior to the development of complications and no sequential assessments of fetuin-A concentrations and other covariates (glycemic control, blood lipids etc.) during the follow-up were available. It is unclear to what extent the intermittent development of diabetes might have affected the fetuin-A levels in our study. An existence of a collider bias induced by restriction on incident diabetes cases cannot be ruled out, however, we did not detect any collider bias with other exposures in the same diabetes setting in another study within the EPIC-Potsdam cohort [47]. Moreover, in order to reverse the causal effect through collider bias, the parameters on the collider bias pathway must be large compared with the true causal effect and therefore unlikely to be missed from the analysis [48]. Our study is the first prospective study investigating the risk of microvascular events with pre-diagnostic levels of fetuin-A, and previous prospective studies on fetuin-A and risk of diabetes-associated CVD [21, 22] have not so far examined the modification effects by diabetes duration. Future investigations should therefore evaluate the stability of the associations with sequential fetuin-A measurements pre- and post-diabetes diagnosis and confirm our findings in other ethnicities, as EPIC-Potsdam participants are almost exclusively of European ancestry. Finally, due to the observational character of this study, the observed associations cannot be necessarily considered causal.

## Conclusions

In persons with T2D, lower pre-diagnosis fetuin-A levels were associated with higher risk of vascular complications, notably microvascular complications, independent of established cardiometabolic risk factors. Inappropriately low fetuin-A levels are potentially etiologically involved in the development of microvascular disease in type 2 diabetes.

## Supplementary Information

**Additional file 1. **Expanded Materials & Methods. References [1–6]. **Table S1. **Age- and sex-adjusted correlations between fetuin-A and cardiometabolic risk factors, n=587. **Table S2.** Sensitivity analyses for the associations between baseline fetuin-A concentrations and incident diabetes-related complications, excluding HbA_1c_≥6.5%. **Table S3. **Associations between baseline fetuin-A concentrations and individual microvascular endpoints. **Table S4.** Associations of baseline fetuin-A with incident total complications after diabetes diagnosis stratified by complications load, n=587. **Table S5.** Associations of baseline fetuin-A with micro- and macrovascular disease prior to (n=25) or after (n=243) diabetes diagnosis. **Table S6**. Associations of baseline fetuin-A with incident micro- and macrovascular disease in type 2 diabetes, accounting for specific nutrients and food intake.

## Data Availability

The datasets analyzed in the current study are not publicly available due to data protection regulations. In accordance with German Federal and State data protection regulations, epidemiological data analyses of EPIC-Potsdam may be initiated upon an informal inquiry addressed to the secretariate of the Human Study Center (Office.HSZ@dife.de). Each request will then have to pass a formal process of application and review by the respective PI and a scientific board.

## Abbreviations

CHS: Cardiovascular Health Study
CI: Confidence interval
CKD: Chronic kidney disease
CVD: Cardiovascular disease
ELISA: Enzyme-linked immunosorbent assay
EPIC: European Prospective Investigation into Cancer and Nutrition
FLI: Fatty liver index
HR: Hazard ratio
MR: Mendelian Randomization
NAFLD: Non-alcoholic fatty liver disease
RBS: Rancho Bernardo Study
T2D: Type 2 diabetes
TLR4: Toll-like receptor 4
