# Supplementary material for: Fetuin-A and risk of diabetes-related vascular complications: a prospective study
Source: Cardiovasc Diabetol. 2022 Jan 8;21:6. doi: 10.1186/s12933-021-01439-8 (PMC8742328; doi:10.1186/s12933-021-01439-8)
Supplement: Supplementary file 1 — Additional file 1. Expanded Materials & Methods. References [1–6]. Table S1. Age- and sex-adjusted correlations between fetuin-A and cardiometabolic risk factors, n=587. Table S2. Sensitivity analyses for the associations between baseline fetuin-A concentrations and incident diabetes-related complications, excluding HbA1c≥6.5%. Table S3. Associations between baseline fetuin-A concentrations and individual microvascular endpoints. Table S4. Associations of baseline fetuin-A with incident total complications after diabetes diagnosis stratified by complications load, n=587. Table S5. Associations of baseline fetuin-A with micro- and macrovascular disease prior to (n=25) or after (n=243) diabetes diagnosis. Table S6. Associations of baseline fetuin-A with incident micro- and macrovascular disease in type 2 diabetes, accounting for specific nutrients and food intake. [file 12933_2021_1439_MOESM1_ESM.docx]

**Additional file 1**

**Expanded Materials & Methods**

*Fatty liver index calculation*

Fatty liver index (FLI) was calculated applying the formula by Bedogni et al. ^1^:

$$FLI=\frac{e^{0.953*\ln\left( TG \right)+0.139*BMI+0.718*\ln\left( GGT \right)+0.053*WC-15.745}}{1+e^{0.953*\ln\left( TG \right)+0.139*BMI+0.718*\ln\left( GGT \right)+0.053*WC-15.745}}*100$$

where TG=triglycerides, BMI=body mass index, GGT=γ-glutamyl-transferase, WC=waist circumference. A FLI ≥60 rules in fatty liver with a good diagnostic accuracy (AUC=0.85, 95% CI: 0.81; 0.88) ^1^.

*Genotyping*

The DNA was extracted from buffy coats using the chemagic DNA Buffy Coat Kit on a Chemagic Magnetic Separation Module I (PerkinElmer chemagen Technologies, Baesweiler, Germany) according to the manufacturer’s manual. Samples from the EPIC-Potsdam participants were genotyped with three different genotyping arrays: Human660W-Quad_v1_A, HumanCoreExome-12v1-0_B (two datasets) and Illumina InfiniumOmniExpressExome-8v1-3_A DNA Analysis BeadChip. Genotyping and quality control of the Human660W-Quad_v1_A and HumanCoreExome-12v1-0_B chips was described elsewhere ^2^. Genotyping using the Illumina InfiniumOmniExpressExome-8v1-3_A DNA Analysis BeadChip was performed in the Life and Brain Center in Bonn, Germany. Detailed description of genotyping and quality control and imputation was previously published ^3^. Briefly, pre-/phasing and imputation were conducted using Eagle2 ^4^ and the Michigan Imputation Service ^5^ with The Haplotype Reference Consortium (release 1.1) as reference panel ^6^. Imputation was conducted in four separate datasets (one for each chip or two for the HumanCoreExome-12v1-0_B chip) using minimac3 ^5^. Imputed files were merged, keeping the minimal R^2^ score from the four files. SNPs were filtered by R^2^ keeping those with values bigger than 0.6.

**References**

1. Bedogni G, Bellentani S, Miglioli L, Masutti F, Passalacqua M, Castiglione A, Tiribelli C. The fatty liver index: A simple and accurate predictor of hepatic steatosis in the general population. *BMC Gastroenterol*. 2006;6:33
2. Langenberg C, Sharp SJ, Franks PW, Scott RA, Deloukas P, Forouhi NG, Froguel P, Groop LC, Hansen T, Palla L, Pedersen O, Schulze MB, Tormo MJ, Wheeler E, Agnoli C, Arriola L, Barricarte A, Boeing H, Clarke GM, Clavel-Chapelon F, Duell EJ, Fagherazzi G, Kaaks R, Kerrison ND, Key TJ, Khaw KT, Kroger J, Lajous M, Morris AP, Navarro C, Nilsson PM, Overvad K, Palli D, Panico S, Quiros JR, Rolandsson O, Sacerdote C, Sanchez MJ, Slimani N, Spijkerman AM, Tumino R, van der AD, van der Schouw YT, Barroso I, McCarthy MI, Riboli E, Wareham NJ. Gene-lifestyle interaction and type 2 diabetes: The epic interact case-cohort study. *PLoS Med*. 2014;11:e1001647
3. Jager S, Cuadrat R, Hoffmann P, Wittenbecher C, Schulze MB. Desaturase activity and the risk of type 2 diabetes and coronary artery disease: A mendelian randomization study. *Nutrients*. 2020;12
4. Loh PR, Palamara PF, Price AL. Fast and accurate long-range phasing in a uk biobank cohort. *Nat Genet*. 2016;48:811-816
5. Das S, Forer L, Schonherr S, Sidore C, Locke AE, Kwong A, Vrieze SI, Chew EY, Levy S, McGue M, Schlessinger D, Stambolian D, Loh PR, Iacono WG, Swaroop A, Scott LJ, Cucca F, Kronenberg F, Boehnke M, Abecasis GR, Fuchsberger C. Next-generation genotype imputation service and methods. *Nat Genet*. 2016;48:1284-1287
6. McCarthy S, Das S, Kretzschmar W, Delaneau O, Wood AR, Teumer A, Kang HM, Fuchsberger C, Danecek P, Sharp K, Luo Y, Sidore C, Kwong A, Timpson N, Koskinen S, Vrieze S, Scott LJ, Zhang H, Mahajan A, Veldink J, Peters U, Pato C, van Duijn CM, Gillies CE, Gandin I, Mezzavilla M, Gilly A, Cocca M, Traglia M, Angius A, Barrett JC, Boomsma D, Branham K, Breen G, Brummett CM, Busonero F, Campbell H, Chan A, Chen S, Chew E, Collins FS, Corbin LJ, Smith GD, Dedoussis G, Dorr M, Farmaki AE, Ferrucci L, Forer L, Fraser RM, Gabriel S, Levy S, Groop L, Harrison T, Hattersley A, Holmen OL, Hveem K, Kretzler M, Lee JC, McGue M, Meitinger T, Melzer D, Min JL, Mohlke KL, Vincent JB, Nauck M, Nickerson D, Palotie A, Pato M, Pirastu N, McInnis M, Richards JB, Sala C, Salomaa V, Schlessinger D, Schoenherr S, Slagboom PE, Small K, Spector T, Stambolian D, Tuke M, Tuomilehto J, Van den Berg LH, Van Rheenen W, Volker U, Wijmenga C, Toniolo D, Zeggini E, Gasparini P, Sampson MG, Wilson JF, Frayling T, de Bakker PI, Swertz MA, McCarroll S, Kooperberg C, Dekker A, Altshuler D, Willer C, Iacono W, Ripatti S, Soranzo N, Walter K, Swaroop A, Cucca F, Anderson CA, Myers RM, Boehnke M, McCarthy MI, Durbin R. A reference panel of 64,976 haplotypes for genotype imputation. *Nat Genet*. 2016;48:1279-1283

**Tables**

**Table S1.** Age- and sex-adjusted correlations between fetuin-A and cardiometabolic risk factors, n=587.

| Cardiometabolic risk factors and biomarkers | **n** | ***r*** | P Value |
| --- | --- | --- | --- |
| BMI, kg/m^2^ | 587 | 0.08 | 0.06 |
| Waist circumference, cm | 587 | 0.08 | 0.05 |
| Alcohol consumption, g/d | 587 | -0.05 | 0.20 |
| Physical activity level, h/week | 587 | -0.12 | <0.01 |
| SBP, mmHg | 539 | 0.02 | 0.63 |
| DBP, mmHg | 539 | 0.02 | 0.61 |
| FLI | 587 | 0.08 | 0.05 |
| eGFR, mL/min/1.73m^2^ | 578 | -0.01 | 0.78 |
| HbA1c, % | 571 | 0.10 | 0.02 |
| Total cholesterol, mg/dL | 587 | 0.10 | 0.02 |
| Non-HDL-cholesterol, mg/dL | 587 | 0.08 | 0.04 |
| Triglycerides, mg/dL | 587 | 0.01 | 0.84 |
| HDL, mg/dL | 587 | 0.14 | <0.01 |
| CRP, mg/dL | 586 | 0.05 | 0.21 |
| Creatinine, mg/dL | 578 | 0.01 | 0.86 |
| Adiponectin, ng/mL | 581 | 0.02 | 0.64 |
| Circulating ALA (18:3 (ω−3)) | 583 | -0.16 | <0.0001 |
| Circulating EPA (20:5 (ω−3)) | 583 | -0.18 | <0.0001 |
| Circulating DPA (22:5 (ω−3) ) | 583 | -0.23 | <0.0001 |
| Circulating DHA (22:6 (ω−3)) | 583 | -0.17 | <0.0001 |
| Circulating palmitic acid (16:0) | 583 | 0.20 | <0.0001 |
| High-fat dairy, g/d | 583 | -0.04 | 0.35 |
| Low-fat dairy, g/d | 583 | 0.04 | 0.36 |
| Coffee, g/d | 583 | -0.03 | 0.44 |
| Total energy intake, kJ/d | 583 | -0.05 | 0.24 |

Sex- and age-adjusted Spearman’s rank correlation coefficients. BMI=body mass index, SBP=systolic blood pressure, DBP=diastolic blood pressure, HbA1c=hemoglobin A_1c_, HDL=high density lipoprotein, CRP=reactive protein C, eGFR=estimated glomerular filtration rate estimated with Chronic Kidney Disease Epidemiology Collaboration group (CKD-EPI) formula, FLI=fatty liver index, ALA=α-linolenic acid, EPA=eicosapentaenoic acid, DPA=docosapentaenoic acid, DHA=docosahexaenoic acid.

**Table S2.** Sensitivity analyses for the associations between baseline fetuin-A concentrations and incident diabetes-related complications, excluding HbA_1c_≥6.5%.

|  | **Total vascular complications** | | **Microvascular complications** | | **Macrovascular complications** | |
| --- | --- | --- | --- | --- | --- | --- |
|  | **N cases/ total N** | **HR (95% CI) per 1 SD (0.06 g/L) difference in fetuin-A** | **N cases/ total N** | **HR (95% CI) per 1 SD (0.06 g/L) difference in fetuin-A** | **N cases/ total N** | **HR (95% CI) per 1 SD (0.06 g/L) difference in fetuin-A** |
| **All participants** | 243/587 | 0.86 (0.74; 0.99) | 203/577 | 0.84 (0.71; 0.98) | 60/587 | 0.92 (0.68; 1.24) |
| **Excluding baseline HbA_1c_≥6.5%** | 155/410 | 0.80 (0.67; 0.97) | 127/403 | 0.76 (0.61; 0.95) | 40/410 | 0.86 (0.55; 1.35) |

**Table S3.** Associations between baseline fetuin-A concentrations and individual microvascular endpoints.

|  | **Nephropathy** | | **Neuropathy** | | **Retinopathy** | |
| --- | --- | --- | --- | --- | --- | --- |
|  | **N cases/ total N** | **HR (95% CI) per 1 SD (0.06 g/L) difference in fetuin-A** | **N cases/ total N** | **HR (95% CI) per 1 SD (0.06 g/L) difference in fetuin-A** | **N cases/ total N** | **HR (95% CI) per 1 SD (0.06 g/L) difference in fetuin-A** |
| **The respective event occurs as 1^st^ event or sole event** | 75/577 | 0.90 (0.70; 1.17) | 79/577 | 0.84 (0.65; 1.08) | 15/577 | 0.91 (0.38; 2.18) |

Associations were assessed by Cox proportional hazards models, are censored at 1^st^ respective event and are shown per one unit SD increase (0.06 g/L) in baseline fetuin-A concentration and adjusted for age at diabetes diagnosis (underlying time scale), duration between recruitment and diabetes diagnosis, sex, education (3 categories: no or in vocational training, vocational training/technical school, technical college or university), alcohol intake (6 categories: <6.1 g/day, 6.1-12.0 g/day, 12.1-24.0 g/day, 24.1-60.0 g/day, 60.1-96.0 g/day, >96.0 g/day), smoking (4 categories: never smoker, former smoker, current smoker <20 cigarettes/day, current heavy smoker ≥20 cigarettes/day), physical activity (sports ≤4 h/week, sports >4 h/week, biking <2.5 h/week, biking 2.5-4.9 h/week, biking ≥5 h/week), body mass index, waist circumference, history of hypertension, hyperlipidemia, antihypertensive and lipid lowering medications at the time of recruitment. Microvascular complications were defined as new-onset retinopathy, nephropathy, neuropathy or kidney replacement therapy. Macrovascular complications were defined as newly diagnosed myocardial infarction or stroke following diabetes diagnosis.

HR=hazard ratio, CI=confidence interval, SD=standard deviation.

**Table S4**: Associations of baseline fetuin-A with incident total complications after diabetes diagnosis stratified by complications load, n=587

| **1 complication** | | **>1 complication** | |
| --- | --- | --- | --- |
| **N cases/ total N** | **HR (95% CI) per 1 SD (0.06 g/L) difference in fetuin-A** | **N cases/ total N** | **HR (95% CI) per 1 SD (0.06 g/L) difference in fetuin-A** |
| 162/587 | 0.82 (0.69; 0.98) | 81/587 | 0.95 (0.73; 1.23) |

Associations were assessed by Cox proportional hazards models and are shown per one unit SD increase in baseline fetuin-A concentrations and adjusted for age at diabetes diagnosis (underlying time scale), duration between recruitment and diabetes diagnosis, sex, education (3 categories: no or in vocational training, vocational training/technical school, technical college or university), alcohol intake (6 categories: <6.1 g/day, 6.1-12.0 g/day, 12.1-24.0 g/day, 24.1-60.0 g/day, 60.1-96.0 g/day, >96.0 g/day), smoking (4 categories: never smoker, former smoker, current smoker <20 cigarettes/day, current heavy smoker ≥20 cigarettes/day), physical activity (sports ≤4 h/week, sports >4 h/week, biking <2.5 h/week, biking 2.5-4.9 h/week, biking ≥5 h/week), body mass index, waist circumference, history of hypertension, hyperlipidemia, antihypertensive and lipid lowering medications at the time of recruitment.

**Table S5**: Associations of baseline fetuin-A with micro- and macrovascular disease prior to (n=25) or after (n=243) diabetes diagnosis.

|  | | **Total vascular complications** | | | **Microvascular complications** | | | **Macrovascular complications** | |
| --- | --- | --- | --- | --- | --- | --- | --- | --- | --- |
|  | **N cases/ total N** | | **HR (95% CI) per 1 SD (0.06 g/L) difference in fetuin-A** | **N cases/ total N** | | **HR (95% CI) per 1 SD (0.06 g/L) difference in fetuin-A** | **N cases/ total N** | | **HR (95% CI) per 1 SD (0.06 g/L) difference in fetuin-A** |
| **Incident complications prior to diabetes diagnosis*** | 25/369 | | 0.98 (0.54; 1.78) | 10/367 | | not enough cases | 23/370 | | 0.93 (0.51; 1.70) |
| **Incident complications after diabetes diagnosis**^†^ | 243/587 | | 0.86 (0.74; 0.99) | 203/577 | | 0.84 (0.71; 0.98) | 60/587 | | 0.92 (0.68; 1.24) |
| **All incident complications*** | 268/612 | | 0.91 (0.80; 1.04) | 213/600 | | 0.89 (0.76; 1.03) | 83/613 | | 0.94 (0.75; 1.18) |

Associations were assessed by Cox proportional hazards models and are shown per one unit SD increase in baseline fetuin-A concentrations.

*Adjusted for age at EPIC-Potsdam recruitment time (underlying time scale), sex, education (3 categories: no or in vocational training, vocational training/technical school, technical college or university), alcohol intake (6 categories: <6.1 g/day, 6.1-12.0 g/day, 12.1-24.0 g/day, 24.1-60.0 g/day, 60.1-96.0 g/day, >96.0 g/day), smoking (4 categories: never smoker, former smoker, current smoker <20 cigarettes/day, current heavy smoker ≥20 cigarettes/day), physical activity (sports ≤4 h/week, sports >4 h/week, biking <2.5 h/week, biking 2.5-4.9 h/week, biking ≥5 h/week), body mass index, waist circumference, history of hypertension, hyperlipidemia, antihypertensive and lipid lowering medications at the time of recruitment.

^†^Adjusted for age at diabetes diagnosis (underlying time scale), sex, education (3 categories: no or in vocational training, vocational training/technical school, technical college or university), alcohol intake (6 categories: <6.1 g/day, 6.1-12.0 g/day, 12.1-24.0 g/day, 24.1-60.0 g/day, 60.1-96.0 g/day, >96.0 g/day), smoking (4 categories: never smoker, former smoker, current smoker <20 cigarettes/day, current heavy smoker ≥20 cigarettes/day), physical activity (sports ≤4 h/week, sports >4 h/week, biking <2.5 h/week, biking 2.5-4.9 h/week, biking ≥5 h/week), body mass index, waist circumference, history of hypertension, hyperlipidemia, antihypertensive and lipid lowering medications at the time of recruitment and additionally adjusted for duration between recruitment and diabetes diagnosis.

**Table S6**: Associations of baseline fetuin-A with incident micro- and macrovascular disease in type 2 diabetes, accounting for specific nutrients and food intake.

|  | **Total vascular complications** | | **Microvascular complications** | | **Macrovascular complications** | |
| --- | --- | --- | --- | --- | --- | --- |
|  | **N cases/ total N** | **HR (95% CI) per 1 SD (0.06 g/L) difference in fetuin-A** | **N cases/ total N** | **HR (95% CI) per 1 SD (0.06 g/L) difference in fetuin-A** | **N cases/ total N** | **HR (95% CI) per 1 SD (0.06 g/L) difference in fetuin-A** |
| Model 1 | 243/587 | 0.86 (0.74; 0.99) | 203/577 | 0.84 (0.71; 0.98) | 60/587 | 0.92 (0.68; 1.24) |
| Model 1 + total dairy | 243/587 | 0.86 (0.74; 0.99) | 203/577 | 0.84 (0.72; 0.99) | 60/587 | 0.93 (0.69; 1.24) |
| Model 1 + coffee | 243/587 | 0.86 (0.75; 1.00) | 203/577 | 0.85 (0.72; 0.99) | 60/587 | 0.94 (0.70; 1.26) |
| Model 1 + omega3 | 242/583 | 0.87 (0.76; 1.01) | 202/573 | 0.86 (0.73; 1.01) | 60/583 | 0.96 (0.71; 1.30) |
| Model 1 + palmitic acid | 242/583 | 0.87 (0.76; 1.01) | 202/573 | 0.85 (0.73; 1.00) | 60/583 | 0.95 (0.71; 1.29) |

Model 1 is the basic model adjusted for age at diabetes diagnosis (underlying time scale), duration between recruitment and diabetes diagnosis, sex, education, alcohol intake, smoking, physical activity, BMI, waist circumference, history of hypertension, hyperlipidemia, antihypertensive and lipid lowering medications at the time of recruitment. Total daily dairy and coffee intake were corrected for total energy intake. Circulating fatty acids were measured in erythrocytes.
